# Cold homes, fuel poverty and energy efficiency improvements: A longitudinal focus group approach

**DOI:** 10.1177/1420326X17703450

**Published:** 2017-04-12

**Authors:** Charlotte N. B. Grey, Tina Schmieder-Gaite, Shiyu Jiang, Christina Nascimento, Wouter Poortinga

**Affiliations:** 1 Welsh School of Architecture, Cardiff University, Cardiff, UK; 2 School of Social Sciences, Cardiff University, Cardiff, UK; 3 School of Psychology, Cardiff University, Cardiff, UK

**Keywords:** Fuel poverty, Cold homes, Energy efficiency, Focus groups, Qualitative

## Abstract

Cold homes and fuel poverty have been identified as factors in health and social inequalities that could be alleviated through energy efficiency interventions. Research on fuel poverty and the health impacts of affordable warmth initiatives have to date primarily been conducted using quantitative and statistical methods, limiting the way how fuel poverty is understood. This study took a longitudinal focus group approach that allowed exploration of lived experiences of fuel poverty before and after an energy efficiency intervention. Focus group discussions were held with residents from three low-income communities before (n = 28) and after (n = 22) they received energy efficiency measures funded through a government-led scheme. The results show that improving the energy efficiency of homes at risk of fuel poverty has a profound impact on wellbeing and quality of life, financial stress, thermal comfort, social interactions and indoor space use. However, the process of receiving the intervention was experienced by some as stressful. There is a need for better community engagement and communication to improve the benefits delivered by fuel poverty programmes, as well as further qualitative exploration to better understand the wider impacts of fuel poverty and policy-led intervention schemes.

## Introduction

### Background

The term *fuel poverty* was coined in the UK following the oil crisis in 1973, a period characterised by a marked increase in fuel prices resulting in many households, particularly those on low incomes, being unable to afford adequate indoor warmth.^1,2^ This underpins the concept of fuel poverty which is most commonly understood as the inability to heat the home to an adequate temperature at a reasonable cost,^3,4^ and is a phenomenon which is now recognised as a significant issue within the UK and across Europe.^5^ Fuel poverty is closely connected to an energy inefficient building stock and is driven by a low household income, fuel prices and under-occupation,^4^ resulting in either low indoor temperatures or a trade-off effect, where warmth comes at the cost of other essentials such as food.^6,7^ This has been linked to adverse effects on physical and mental health, and to negative impacts on social wellbeing.^6,8^ Households on low incomes often live in deprived areas with poor quality housing that is expensive to heat,^9^ and as such, the Marmot Review Team has identified cold homes and fuel poverty as major factors in health and social inequalities that could easily be addressed through energy efficiency interventions.^10^

The UK has one of the oldest existing housing stocks in the EU,^11^ with many houses built in Victorian times. Wales, in particular, has a large proportion of solid-walled properties dating back to its past as a thriving industrial economy of coal and slate mining. These small solid-walled houses make up 32% of total dwellings in Wales, with a higher number in rural areas (37%) compared to urban areas (29%).^12^ In addition, an estimated 20% (264,500) of dwellings in Wales are not connected to the gas network,^13^ with a higher proportion of these in rural areas^12^ and are therefore reliant on more expensive fuel types, such as heating oil, liquefied petroleum gas or electricity. This partially accounts for the high prevalence of fuel poverty in Wales, with an estimated 30% (386,000) of households categorised as fuel poor by Welsh Government in 2012.^14^

The domestic building sector contributes about 11% to the UK’s CO_2_ emissions,^15^ which has led to energy efficiency investments in existing housing stock emerging as a central pillar to tackling fuel poverty and simultaneously reducing carbon emissions.^16^ All considered, it is perhaps not surprising that a number of policy-led programmes, both at national and local level, have been developed with a view to increase energy efficiency in the domestic building stock and simultaneously reduce fuel poverty in some of the hardest hit areas.^17–19^ In 2001, the UK *Fuel Poverty Strategy* was developed and formally adopted a measure of fuel poverty, whereby a household is defined as fuel poor if it needs to spend more than 10% of its income on fuel to maintain an adequate indoor temperature of 18℃ throughout the home and 21℃ in the living room.^20^ In Wales, fuel poverty became a partially devolved issue after 2006, and in 2010, the Welsh Government published the Fuel Poverty Strategy for Wales, maintaining the 10% threshold, and introducing two major policy-led schemes to tackle fuel poverty: Nest, a demand-led scheme, and Arbed, an area-based scheme.^17^

### Fuel poverty, housing quality and health

Hills cautions that there is not necessarily a direct cause and effect between fuel poverty and health, but rather that certain drivers of fuel poverty are linked to living in low indoor temperatures.^4^ Although many of the health consequences stem from prolonged exposure to low indoor temperatures,^4^ other negative effects can arise through the challenges which fuel-poor households face in order to keep warm.^6^ Hills determined that fuel poverty disproportionately affects the elderly, infants and individuals with disability and long-term illness. These vulnerable groups are also likely to have higher than average energy and heating requirements and are prone to spending long periods of time indoors.^4^

Fuel poverty and living in cold and damp conditions can have a negative impact on the occupants’ physical and mental health^10,21^ and can exacerbate existing conditions, such as respiratory or cardiovascular problems.^22–24^ The literature shows that low indoor temperatures are commonly associated with a wide range of negative health consequences, including an increased risk of strokes, heart attacks and respiratory illnesses, as well as with common mental disorders.^10,25^ Moreover, around 40% of excess winter mortality is attributable to living in a cold home.^26^ It has been estimated that the morbidity and mortality associated with living in fuel poverty and cold homes costs the NHS approximately £1.36 billion a year, with further costs associated with social care services and informal care providers.^27^

There are a number of plausible mechanisms linking poor quality housing to different health outcomes and excess winter mortality, such as the strain of thermal stress on the cardio-respiratory system.^28^ Furthermore, cold and damp buildings are often prone to mould which may trigger or exacerbate respiratory conditions through allergy, infection and toxicity.^29^ In terms of mental health, it has been noted that living with thermal discomfort and low temperatures can increase common mental disorders.^30,31^

As well as direct health effects, cold housing and fuel poverty also have indirect impacts on wellbeing and life opportunities.^10^ Reduced emotional wellbeing, social isolation, financial burden causing stress and malnutrition and difficulties staying warm are all socio-economic factors associated with fuel poverty that are affected through spending high proportions of income on heating.^10^ People experiencing fuel poverty have various strategies for coping, either through (1) rationing to try and limit fuel bills; (2) making difficult financial decisions on household expenditure; or (3) adopting neither of the two previous approaches, increasing the risk of debt and disconnection.^32^ Rationing could take place by heating only the main living room thereby limiting usable space in the home, known as *spatial shrink*, which can result in social problems for the household including a lack of privacy and poor outcomes for young people.^33^ Additionally, the use of heating may be temporally restricted, causing a cold home and thermal discomfort. Those living in fuel poverty who choose to prioritise warmth are forced to make difficult decisions about household essentials which can lead to poor diets, known as the *heat-or-eat* dilemma, withdrawal from the community, social isolation due to a reluctance to invite people into the home or the inability to go out and socialise and financial stress.^4^ The third approach may lead to fuel-poor households continuing their normal spending patterns on fuel and other items, which could result in arrears in fuel payments and the accumulation of other types of debts.^34^

It has been stressed that energy efficiency measures and interventions are the main and simplest ways of tackling fuel poverty and preventing its associated physical health and socio-economic consequences,^10^ as well as protecting against deteriorating mental health.^35^ Thomson et al.^36^ undertook a systematic review of warmth and energy efficiency intervention studies and concluded that improvements in general health, respiratory health and mental health could be seen in several studies, although the overall impact was not clear in part due to the variety of intervention measures and limited follow-up periods. Studies targeting specific groups with existing chronic respiratory conditions showed the greatest improvements of symptoms following the energy efficiency intervention.^21,37^ Difficulties in observing changes in health resulting from energy efficiency interventions could also be linked to the complexity of fuel poverty and differences in the ways people experience it, supporting the need for qualitative and mixed method approaches to the study of fuel poverty and interventions.

### The need for qualitative research

It is clear from the literature that fuel poverty has primarily been examined by means of quantitative and statistical methods, and that to date only a limited number of studies on qualitative aspects of fuel poverty and energy efficiency improvements have been conducted.^36^ Much of the research has focused on quantitative data looking at key health outcomes to try and establish a causal pathway to health from cold homes.^21,36^ However, fuel poverty is a complex social issue that stretches far beyond a simple model of cause and effect. For instance, fuel poverty is measured at an individual level but tends to have broader repercussions for households as a whole, as well as for neighbourhoods and the wider community. One issue is that a cause and effect arising from housing improvements are likely to only be measurable after a longer period of time.^38^ Further, there is a need for a move towards looking at housing and the immediate environment in a broader sense, as well as a better understanding of the complex socio-economic factors and the individual experiences all potentially affecting the health and wellbeing of occupants. There are only a small number of studies overall which have explored the social and socio-economic impacts of housing improvements.^36^ There is a need to understand the broader social and emotional outcomes linking housing interventions to physical and mental health,^21^ particularly through qualitative studies aiming to explore the lived experience of those living in fuel poverty^39^ and also to examine the impacts of the intervention process on the recipient.^38^

Fuel poverty is a concern for several policy spheres including poverty, health inequalities, health service use and carbon emissions, as well as for the health and wellbeing, thermal comfort, financial stress and social interactions of affected households.^4^ Due to this multifaceted nature of the problem, different methodological approaches are needed to try and understand fuel poverty from a number of angles and perspectives, and particularly from the view of the affected households. To understand the wider picture of what is going on, there is a need to include methods of unstructured and open-ended data collection that allow a topic to be explored in greater depth.^40^ Further, qualitative research is essential in order to understand how change is experienced in the daily lives of the fuel poor.^39^ With research areas such as fuel poverty, which is caused by a number of interrelated factors and touches upon many different issues, a more flexible research approach is imperative to allow themes to emerge from the data. Since a more rigid, quantitative approach can lead to important issues being overlooked due to them not being considered in the initial research catalogue, longitudinal focus groups were chosen as a study design. Longitudinal focus groups facilitate open group discussion and dynamics to ensure flexibility throughout, in order to explore the broader experiences of recipients of an intervention at different stages of the process.

### This study

#### Aims of the study

The study had two key aims. The first aim was to obtain a better understanding of the views and experiences of low-income households who were at an increased risk of fuel poverty through living in cold, energy inefficient (*hard-to-heat, hard-to-treat*) houses. The second aim of the study was to explore the application of a longitudinal focus group approach as a qualitative method to explore the ways in which experiencing an energy efficiency intervention changed the views and experiences of residents. To date, only a small number of studies have been conducted to examine qualitative aspects of warmth and energy efficiency improvements, and the majority of these were individual interviews at a single point in time. To our knowledge, no repeated focus groups have been conducted to explore the ways in which energy efficiency improvements may change residents’ experiences of previously living in cold, energy inefficient homes and to better understand their personal and detailed experiences of receiving energy efficiency work through a large-scale, policy-led intervention scheme. The qualitative study is part of wider research examining the health impacts of structural energy-performance investments using quantitative and monitoring methodologies,^41^ and builds and expands on the same topics investigated using quantitative techniques within a subset of the same participants.

#### The intervention programme

In this paper, we focus on the Welsh Government’s strategic energy performance investment programme (*Arbed phase 2*) which was created with the three-pronged aim of (1) reducing the number of households living in fuel poverty, (2) creating jobs and regeneration in Wales and (3) combating climate change by reducing household energy use. Social interventions, such as the Arbed programme, differ from clinical and more complex public health interventions in that changes in health are often an indirect effect rather than a primary aim of the intervention.^42^ The programme took place between 2012 and 2015 and targeted mixed-tenure houses in selected low-income, communities identified as being at risk of fuel poverty. Households within these areas were eligible to receive multiple energy efficiency measures to improve the energy performance of their homes free of charge and without means-testing.^43^ Typical energy efficiency measures included external wall insulation, central heating system upgrades and connecting a community to the mains gas network. As an area-based programme, measures were provided on a street-by-street or community basis. For a typical household, the process of the energy efficiency work being undertaken would generally have taken at least a few months, depending on the type of measures selected for that scheme. The work was undertaken on a community basis to reduce overall cost, with each household being in contact with a variety of stakeholders from community engagement officers, to project managers and contractors.

## Methods

### The focus groups

The longitudinal focus group study consisted of six meetings in three pre-selected case study areas, chosen from communities that were due to receive energy efficiency work and who were also participating in the quantitative and monitoring parts of the wider study project.^41^ It is the results of the focus groups which are reported here.

The first series of focus group discussions took place just after winter had finished and before the intervention work was conducted. The same participants were invited to take part in the second round of focus group discussions, that took place the following spring after all improvement work had been completed and a heating season had been experienced. Box 1 provides the details of each community as well as specifics of the three schemes included in the research. The first round of focus groups was held in March 2014 and lasted just under 1.5 h. In total, 28 people took part in the study (eight in Caerau, nine in Brynamman and 11 in Hollybush). Participants were recruited from three selected communities, where the monitoring study had taken place as part of the wider project.^44^ Care was taken to avoid pre-selection of individual participants for the groups due to the risk of introducing researcher bias. The focus groups were held at a convenient location and took place either at lunchtime or in the early evening. The second round of focus groups was held in March and April 2015, and again lasted just under 1.5 h. All of the participants who took part in the first round were invited to attend. In total, 22 people took part in the reconvened focus groups (five in Caerau, three in Brynamman, and 14 in Hollybush). This included three additional residents from the Hollybush area who expressed interest in attending the discussions.

**Box 1.** Description of the communities and interventions.

**Caerau** is a suburb located three miles to the west of Cardiff city centre. Housing is a mixture of housing association flats, bricked terraced houses, traditionally built semi-detached houses, and semi-detached BISF (steel framed) houses. The intervention work that took place here was external wall insulation (EWI) and boiler/heating system upgrades. Caerau has a Welsh Index of Multiple Deprivation (WIMD)^a^ rank of 170 and is part of the Cardiff West Communities First regeneration cluster.
**Brynamman** is a village located on the south facing side of the Black Mountain, in an old coal mining area within the Brecon Beacons National Park boundaries. The area is currently off the mains gas network and contains mainly small stone terraced houses. The intervention work extended the mains gas network to homes in the village, and provided boiler upgrades. The area has a WIMD score of 651.
**Hollybush** is an old coal mining village situated between Blackwood and Tredegar, above the Sirhowy Valley. The area was off the mains gas network, and contains a combination of older small stone terraced houses (pre-1919) and post 1965 and 1980s detached homes. The intervention work extended the mains gas network to homes in the village and provided boiler upgrades. The area has a WIMD score of 565, and located within the Mid Valleys East Communities First regeneration cluster.

a WIMD scores range from 1 (most deprived) to 1909 (least deprived).

Unstructured topics for the focus group discussions were loosely based on the themes from the household survey used for the community-based quantitative study.^41^ The same themes were used to guide the discussions in the before and after focus groups, and broadly covered the areas of (1) health and wellbeing, (2) thermal comfort, staying warm, and the use of living space, (3) fuel poverty and (4) experiences with the intervention programme. Participants were free to introduce and discuss other topics throughout. The role of the moderator was to intervene minimally, but to guide the themes for conversation where needed. Ethical approval was received from the Welsh School of Architecture’s Research Ethics Committee on 15 March 2014 (EC1403.184). Participants were offered a £30 voucher as compensation for their time.

### Analyses

**Figure 1.**
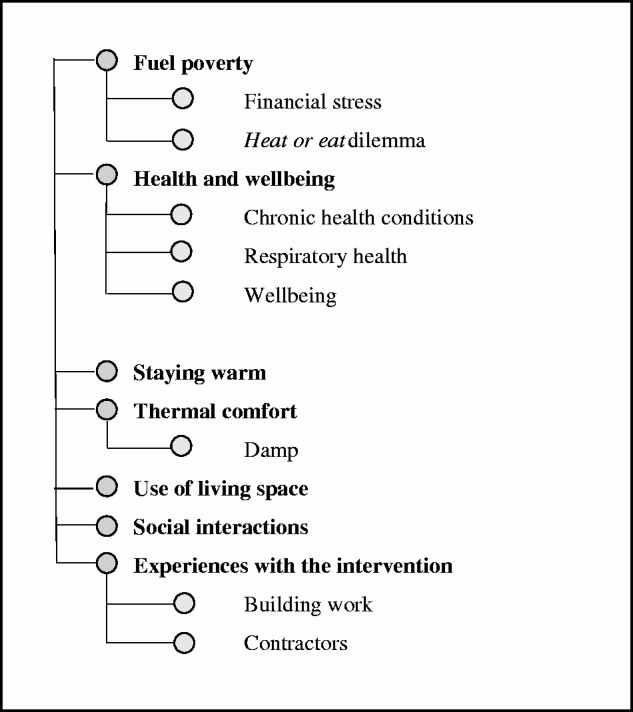
Coding tree with coding parent and child nodes.

The dynamics of focus groups leads to a large quality of detailed information. Compared to one-to-one qualitative interviews, the interaction between participants in the group allows issues to be probed and views challenged or modified. To understand the detailed data collected, a structured approach was applied for the analysis. The focus groups were recorded, transcribed and coded using computer-assisted qualitative data analysis software. NVivo was used to code the transcripts using the key topics, described above, which were based on identified themes within the context of the existing fuel poverty literature and policies. Transcripts were coded separately by two researchers to ensure consistency. Figure 1 shows the coding tree that was used to analyse the focus groups’ transcripts. The same coding scheme was used for both waves of focus groups in order to examine the changes following the intervention. After coding, each parent and child node was analysed thematically and emerging patterns were refined and cross-compared. NVivo was used to count the frequency that key terms were discussed before and after the participants received the intervention.

## Results and discussion

Choosing the format of a focus group allowed the participants to express in their own words how they perceived their homes and their health before and after the intervention, as well as their experiences with the intervention programme, in as much detail as they wanted. This provided a much more detailed and broad spread of information than can normally be collected by means of a quantitative survey. To provide a balanced view of focus groups as a qualitative method of data collection, it is worth looking at both the negatives and the positives, and being aware that the group environment can have the poterntial for unwillingness to discuss subjects that might be considered personal or stigmatising. Although it was made clear that all information recorded would be anonymised. Most importantly, though, the focus groups have allowed the participants to have a voice on fuel poverty and to discuss and explore the issues they deemed significant. Participants appeared keen to share their experiences, and the group dynamic allowed valuable understanding of why people feel the way they do.^45^

### Descriptive findings

An initial word frequency analysis of the pre- and post-intervention transcripts showed a move away from terminology and phrasing related to thermal discomfort after the energy efficiency measures had been installed. Table 1 shows a comparison of a number of relevant words and their occurrences in the focus groups. The frequency with which certain themes appear between the two focus group events gives an indication of topics on people’s minds and how priorities in their lives might have changed as a result of the energy efficiency measures. While these transcripts cannot be seen in isolation, and provide a snapshot of what can be considered a very dynamic issue, it is noteworthy that words associated with heat, warmth, cold and insulation have been used significantly less after the measures had been installed. ‘Damp’ in particular had been a concern for many participants in the first round of focus groups, with 39 occurrences in the conversations. By the time of the second focus group’s session, it featured only eight times in total.

**Table 1.** Comparison of word frequency of key terms pre-and post-intervention.

Word	Frequency pre-intervention	Frequency post-intervention
Heating	107	79
Cold	97	46
Insulation	70	40
Warm	66	24
Money	59	75
Damp	39	8
Arbed	40	58
Bill	33	33
Comfortable	32	34
Health	28	27
Total	571	424

### Thematic findings

Here, the results and findings from the two focus groups are presented in the following key themes: health and wellbeing; thermal comfort and staying warm; use of living space and social interactions and fuel poverty, including the ‘*heat-or-eat*’ dilemma and financial stress, with anonymised quotes from the discussions.

#### Health and wellbeing

The adverse effects of fuel poverty and cold homes on health are well documented,^4^ but it is important to remember that ill health can be both an effect and cause of fuel poverty,^39^ where householders have to trade-off exacerbation of health conditions from the cold with increased costs of maintaining a satisfactory temperature inside their home. During the *pre-intervention* focus groups, most participants agreed that their health and wellbeing was detrimentally affected by living in a cold home. This covered both mental and physical health aspects. In line with Harrington et al.,^8^ participants felt that living in a cold home exacerbates ill health rather than causing it. While respiratory conditions were most frequently mentioned as being exacerbated and prolonged by cold and damp living conditions, other health conditions like diabetes, arthritis, Raynaud’s disease and circulatory issues were also perceived to increase in severity. In particular, participants thought that a cold home may make it more difficult to live with or recover from pre-existing chronic conditions. As was stated by one participant: ‘It’s a fact that I suffer from a lung disease and it would be better if I were in somewhere warmer.’ (Male, pre-intervention)

*Post-intervention*, health concerns seemed to have decreased and general wellbeing was perceived to have been improved, in line with findings by Gilbertson et al.^46^ As one participant noted: ‘I suffer with Raynaud’s disease in my hands and feet as well so keeping those warm is vital really and last winter it was a nightmare whereas this winter has been a bit better.’ (Female, post-intervention)

In terms of mental health, participants felt, *pre-intervention*, that living in a cold home may contribute to poor emotional wellbeing. The physical effects of exposure to poor internal conditions, as well as psychological stress resulting from heating bills were felt to trigger feelings of being ‘miserable’, ‘depressed’, ‘anxious’, but also of feeling ‘ridiculous’ with regard to the extent of measures taken to staying warm. *Post-intervention*, general mental health appeared to have improved among participants, with one respondent mentioning:

For me certainly, I don’t have to sit snuggled up in a blanket every night now so I can sit comfortably as a general rule. So from that view point it is more comfortable and obviously that affects your state of mental health, if you’re going to sit more and watch television or listen to music or whatever, it’s certainly more comfortable. (Female, post-intervention)

It was generally believed that a warmer home environment would contribute to better mental and physical wellbeing. This was frequently reflected in the choice of words participants used to describe how they felt *post-intervention* such as ‘lovely’, ‘really nice’ and ‘comfortable’. Generally, there was a correlation between feeling adequately warm and being relatively healthy and active: ‘If you are warm, you do more, you want to do more, otherwise you just want to sit and huddle.’ (Female, post-intervention)

Energy efficiency measures could also have an impact on the exterior of the buildings with insulation and new rendering improving the aesthetics of the homes, the positive effects of which were observed by the respondents: ‘If the sun’s out you feel happy. If your house is nice you feel happy.’ (Male, post-intervention)

It is noteworthy that health and wellbeing were not discussed in as much detail *post-intervention* as in the *pre-intervention* round of focus groups. It appeared that health had become a lower priority, and it proved more labourious to sustain discussions on the topic. While the term ‘health’ was mentioned as frequently in the post-intervention as in the pre-intervention focus groups in terms of prevalence from the word frequency analysis (see Table 1), the topic took a much less prominent role in the discussions. Where people discussed the topic, they suggested that better thermal comfort and the aesthetics aspect of the improvements had been beneficial for their general feelings of wellbeing. ‘People come down see and says that looks lovely, and that’s bring you up …’ (Male, post-intervention)

#### Thermal comfort and staying warm

When asked about the thermal conditions in their homes during the colder seasons, *pre-intervention* discussions showed a strong choice of words such as ‘suffering’, ‘horrendous’ and ‘freezing’ combined with the use of qualifiers such as ‘absolutely’ and ‘all the time’. Participants described how difficult it is to stay warm in an energy inefficient house, in particular if it is non-traditional housing (e.g. steel-framed construction), even if heating was being used: ‘I’m freezing! Literally, in my house all the time.’ (Male, pre-intervention)

As a result, they devised and employed a number of strategies to stay warm and avoid having to turn up the heating. This included the use of portable heaters, hot water bottles and blankets and electric blankets, or only heating certain rooms in the house. It is notable that these strategies involve the heating of individuals rather than spaces:

I used to wake up to Jack Frost in the morning and it wasn’t unusual to go to bed with a scarf round your neck. My electric blanket has saved me this winter. That was the best thing I ever bought was my electric blanket! Give everybody an electric blanket! (Female, pre-intervention)

Similar strategies were reported by participants in three other qualitative studies.^8,35,46^ These strategies were, however, often seen as temporary stopgaps used because heating was unaffordable, and as unsustainable in the longer term:

Imagine if you’d come to our house and we were sitting there with throws on, fully dressed and hot water bottles. And during that we’d probably do the hot water bottles twice! Just to sit there and watch television. We were frightened to turn any of the heating on.

*Post-intervention* discussions show that participants perceived their home as warmer and more comfortable and easier to maintain consistent warmth. They acknowledged that this was a positive result of the energy efficiency measures put in place by the intervention programme, namely the external wall insulation and more efficient central heating systems. This meant that they no longer needed to find ways to economise on their heating bills, for example by restricting their use of fuel or finding alternative ways of keeping warm: ‘I definitely noticed that we’re using les*s* electric and gas. Because it’s warmer. It is warmer, end of. You got a two-foot thick blanket around your house, it’s bound to make it warmer.’ (Male, post-intervention)

They discussed that they now could more easily afford to heat their whole house, rather than only a few rooms. This negated the need to use of ‘stay warm’ strategies as mentioned above. The energy efficiency measures not only improved the overall quality of the indoor environment, it also opened up rooms that were previously left unheated and as such used less, effectively increasing the amount of usable living space within the home. Similar to the findings of Harrington et al.,^8^ participants attached great importance to their increased ability to keep their homes warm. Most of the participants stated that their homes were now much warmer during the winter and cheaper to heat. On average, UK temperatures were slightly lower during the second post-intervention winter,^47^ suggesting the energy efficiency measures can explain the reported improvements.

#### Fuel poverty, the heat-or-eat dilemma and financial stress

Financial considerations were a common ground of worry in the *pre-intervention* focus groups. The focus group participants repeatedly mentioned how expensive it is to heat their homes and talked about how easy it is for low-income households living in an energy inefficient house to fall into fuel poverty. The large proportion of off-grid properties with no access to the gas network have to choose more expensive fuel types, including oil or electricity: ‘People are buying oil on credit cards just to keep warm, it is – it is quite sad.’ (Male, pre-intervention)

*Post intervention,* participants in all three focus groups discussed in detail how it had become cheaper and easier to keep their homes warm and comfortable, suggesting that the energy efficiency work had indeed made a substantial difference to their feelings of fuel poverty: ‘We’ve got heating on all the time because I just don’t care about the bills now. I just think I’ll leave it staying on so the house is warm.’ (Female, post-intervention)

For fuel-poor households, high heating bills are not only stressful, they also force householders to make difficult choices in their daily lives.^4,48^ *Pre-intervention*, the ‘*heat-or-eat*’ dilemma was a common factor for focus group participants directly or members of their communities. It was noted that the discomfort of living in a cold and damp home can be exacerbated by inadequate diet and financial stress, creating a cumulative effect:

You know if you are in a cold, damp house and you haven’t got adequate food, then obviously the impact of being in that cold, damp house is going to be far worse than if you had, you know, a really good diet or the ability to go out of that house for a few hours and have a meal in a warm, comfortable environment you know things like that. So I think it is more than just the way you heat your house, I think there is a broader – a broader brush to wellbeing and feelings of wellbeing.’ (Female, post-intervention)

The findings support the conclusions of Harrington et al.^8^ that the health impacts of fuel poverty involve more than the direct physical effects of exposure to poor internal conditions: ‘I wouldn’t eat because we had to heat the house. You know you do think about, it sounds, you think people are heroes who do that. They’re not, they’re everyday people who think oh I can’t do that today.’ (Male, pre-intervention)

Previous studies suggest that improved energy efficiency leading to reduced fuel bills could improve diets.^46,49^ However, in our *post intervention* focus groups, only one participant discussed food specifically and how the ability to save money on heating costs meant having more to spend on diet. ‘She has to have special food that costs more, more than ordinary food, so you save one end …’ (Male, post-intervention)

*Pre-intervention,* participants said they frequently had to make compromises on how to spend their limited household budget. Just as reported by Harrington et al.,^8^ households either economised on their heating bills or refrained from other activities or expenditures in order to stay warm. Compromising has been mentioned as a common coping strategy to deal with fuel poverty. This has been reported by Tod et al.,^50^ who concluded that the need to manage priorities against resources often meant making ‘trade-offs’, that in turn influence behaviour. Fuel poverty forces householders to carefully consider everyday choices and leads to financially cautious, frugal and at times obsessive behaviour: ‘My wife […] she knows exactly what it costs to boil a cup in the kettle.’ (Male, pre-intervention)

During the *pre-intervention* discussion on fuel poverty participants often used very emotive language, often using words such as ‘sad’, ‘hard’, ‘horrendous’, ‘struggle’ or ‘in tears’ to express their struggles. This highlights the level of stress and anxiety that accompanies financial struggles caused, for instance, by high fuel bills: ‘It’s a horrible feeling when you’ve got the gas bill … and you think oh my God, how much is this going to be?’ (Female, pre-intervention)

*Post-intervention*, this association with stress and anxiety was no longer present in the focus group discussions. In contrast to Gilbertson et al.,^46^ who found that householders experienced warmer homes but did not necessarily notice lower heating bills, the focus groups’ participants in this study were acutely aware of their heating costs being lower after the installation of the energy efficiency measures. The type of measures received by different intervention programmes is likely to have varying impacts on fuel bills. All participants seemed unanimously pleased with the decreased financial pressure. Participants spent a large amount of time discussing their financial savings in detail, seeing as their financial constraints had caused them to be acutely aware of their fuel spending before the intervention:

I would say our bills are about a quarter of what they were when we first moved in, we’ve got absolute comfort, peace of mind and economy … Well, just absolutely perfect, it really made the home a proper home rather than a cold house […]. (Female, post-intervention)

In our study, participants who had been connected to the main gas network were particularly happy that they were now able to pay a competitive dual-fuel energy tariff, rather than being dependent on heating oil with highly fluctuating prices. Some of the households had been moved to a main gas supply after having previously used alternative, often more expensive means of heating, such as oil. This had a direct impact on the perceived value of their properties: ‘I’d say it was about £30,000 difference on the house now.’ (Female, post-intervention)

#### Use of living space and social interactions

*Pre-intervention*, participants reported great variations of temperature in different parts of their homes, which had a direct effect on their use of space, making the coldest and therefore most uncomfortable rooms less likely to be used during the heating season. This lack of thermal comfort and living space was thought to put a strain on social interactions within the households and also impacted on social visits from friends and family, and as a result, detrimentally affected participants’ enjoyment of their home. Previous suggestions that fuel poverty and living in a cold home can exacerbate social isolation, both in terms of preventing people from going out as well from inviting others into their home, have been confirmed in the focus groups:^4,10,20^ ‘Living in a house that is cold is miserable. My mum won’t come and visit me.’ (Female, pre-intervention)

This also meant that some householders tried to avoid being at home for certain periods of time altogether. Some of the participants’ responses almost engendered a sense of homelessness, with home being the least preferred option to spend time, if avoidable: ‘I used to prefer to go away to the caravan than stay at home, because I could have the heating on in the caravan the whole time! And that’s how bad it’s been for the heating for us.’ (Female, pre-intervention)

A few participants discussed, *post-intervention*, feeling more comfortable inviting family or friends into their homes, which of course reduced the risk of feeling socially isolated. Participants also noted that they used an increased proportion of space in their homes as a result of the measures. Doors were allowed to remain open, and there was no need to lock off rooms because heating their homes had become more affordable: ‘We got heating on in all the rooms now […] because it’s not such a concern now about the heating bill.’ (Female, pre-intervention)

Similar to the findings in this study, increased useable living space had also been attributed to energy interventions in three other qualitative studies.^8,46,51^ Similarly, energy efficiency interventions have been reported to have a positive impact on privacy, social relationships and space to study^8,46,51^ as well as reducing tension in the household and increasing emotional security.^4,10,46^ Generally, occupants reported a great improvement in how they use the space within their home and how comfortable they are within it, providing further supporting evidence for the findings of the above mentioned studies. This positive attitude and positive change is notable in the choice of words in the focus groups where ‘cold’ had only been discussed half as often *post-intervention* than *pre-intervention*. Similarly, the term ‘damp’ came up only 8 times *post-intervention* compared to 39 times *pre-intervention*.

#### Experiences with the intervention programme

*Pre-intervention*, members of the focus groups welcomed the energy efficiency measures they were expecting to receive under the intervention programme. The participants who had already received some of the measures felt grateful for them, not only because they were provided for free but also because they had made a noticeable difference to their comfort, finances and overall quality of life. These results are in line with the findings of previous qualitative studies. Gilbertson et al.^46^ reported that recipients of Warm Front energy efficiency work were generally positive about the upgrades, and felt that the upgrades had improved thermal comfort, use of living space and feelings of wellbeing. Gilbertson et al.^46^ also found that greater warmth and comfort further enhanced emotional security, social relations within the home and eased symptoms of chronic illness. All of these perceived improvements are reflected in the language chosen during the focus groups, with participants using terms such as ‘grateful’, ‘nice’, ‘absolutely fantastic’, ‘fabulous’, ‘lucky’ and ‘lovely’. ‘[…] we’re having a new central heating system, boiler, radiators, absolutely fantastic! I can’t fault that. You’re having what £5,000 or £6,000 worth of work, for nothing!’ (Male, pre-intervention)

While participants of the first round of focus groups were generally positive about the intervention programme and were looking forward to the improvements, they felt that the communication with the recipients could be improved and households themselves should have a greater say in the delivery of the programme. The ‘one size fits all’ approach was criticised, and many questioned the motives for taking this approach and the usefulness of some of the measures offered. ‘I think some properties need to be looked at on an individual case by case basis. So that you are getting the best fit for your property, not a misfit one size fits all policy.’ (Female, pre-intervention)

Overall, the intervention schemes were generally well-received by householders before the start of the work and are thought to have made a big difference to the warmth of their homes *post-intervention*: ‘As far as the Arbed scheme goes then yes, it’s certainly improved my quality of life. The house is more comfortable and the bills have gone down.’ (Female, post-intervention)

They particularly welcomed that the improvements were provided for free. Participants felt that energy efficiency programmes are very important for low-income communities. The work was not only seen to be beneficial in terms of providing affordable warmth, it was also felt that the external wall insulation had improved the aesthetics of both their homes and their neighbourhoods as a whole. Participants discussed how this had improved feelings of pride in the community as well as general emotional wellbeing: ‘People come down, see and says that looks lovely, and that brings you up … a sunny day makes you happy and looking at your house being nice as well you think, aahh that’s cracking …’ (Male, post-intervention).

The participants were more critical about the delivery of the energy efficiency intervention. They expressed some dissatisfaction with the quality of communications, confusion about the intervention programme and who was delivering it, the quality of work conducted by contractors and a lack of involvement in the selection of energy efficiency measures they felt would be the most beneficial. Some of the occupants who had private or social landlords felt powerless with regard to their role with the programme managers and communication. The results regarding the delivery of the programme resonates well with the findings of previous research, where some residents find the process of installation disempowering and stressful, which could undermine the wellbeing of an already vulnerable population.^38,46,52^

There was scaffolding for a year and a half, wasn’t being used. (Male, post-intervention)

I have to say, I had the team from hell, and I’m still waiting for remedial work and snagging to be done. (Female, post-intervention)

Harrington et al.^8^ argue that people in fuel poverty should not be viewed as passive targets for benevolence and that Government-funded interventions will confer far greater benefits if recipients are made to feel empowered. This was also reflected in the discussions on the delivery of the programme: ‘The thing is though, I think their attitude was – look you’re having it done for nothing, just shut up and take it.’ (Male, post-intervention)

A greater emphasis on involving the individuals can improve feelings of personal control, and as a result, alleviate stress associated with the delivery of a housing improvement programme, which can have a beneficial impact on health outcomes.^38^ Stress has been identified as a significant factor in explaining health inequalities related to poor quality housing.^38,53^ It is important not to underestimate the significance of residents’ views in order to maximise the impact of policy-led programmes.^54^ Indeed, participants of the reconvened focus groups felt that the benefits of the programme would have been greater if they had been more closely involved in the decision-making process. Scott et al.^55^ similarly concluded that the success of the uptake of policy-led energy efficiency schemes is hinged on improved efforts to engage with communities, as well as to listen to and tailor measures to their needs, particularly in deprived communities where there is a reduced willingness to adopt new technologies.

## Conclusion

The longitudinal focus groups showed the importance of improving the energy efficiency of houses at risk of fuel poverty in low-income neighbourhoods. Risk factors for fuel poverty contribute to physical and emotional ill health, and huge financial stress with associated problems of social isolation and the *heat-or-eat* dilemma, particularly in those with pre-existing ill health. The results show clearly the detrimental effect of living in a cold home that is prohibitively expensive to heat because of fuel poverty risk factors, such as energy inefficient homes or expensive fuels. Living in a cold home was viewed as depressing, stressful and detrimental to both mental and physical health, particularly for those with pre-existing ill health. According to the participants, the intervention measures to make the home more energy efficient made great improvements to the comfort and warmth of their homes, opened up spaces within the home and substantially reduced their heating bills. This not only helped to relieve financial stress and fuel poverty it also made them feel less socially isolated. Participants felt that physical health improvements following the work were secondary to improvements in thermal comfort and their ability to invite friends and family into their homes, suggesting that the benefits of the improvements were, at least in the short term, more closely linked to better wellbeing due to broader socio-economic factors related to fuel poverty such as reduced stress from financial pressure, resulting from improved thermal comfort and better control over the heating in their homes, than to the direct physical effects from improved internal temperatures.

A focus on quantitative data collection does not allow for an interpretivist identification and exploration of factors which are important to the recipients of the intervention, or for detailed insight into their varied views and experiences in what is a complex and interdisciplinary subject. Quantitative data collected on fuel poverty and the impacts of energy efficiency and warmth interventions allows for a response from much higher numbers of subjects, and to date the majority of studies have taken up different variations of this approach. In comparison, only a handful of studies have undertaken detailed qualitative exploration of the same topic, and a longitudinal focus group approach is novel in this context.

The advantage of focus groups is that they allow detailed exploration of themes through the ways that individual participants discuss particular issues that they deem important and significant, but within a group dynamic, responding to one another. This enables the researcher to build up a view based on the group interaction.^45^ The results of the focus groups allowed a detailed insight into residents’ experiences of living at risk of fuel poverty, the benefits they perceive from receiving an energy efficiency intervention and their views and experiences of the intervention process, which could only be captured through qualitative exploration. Undertaking a longitudinal collection of data from a series of focus groups allows valuable understanding of the views and lived experiences of individuals living at risk of fuel poverty, and how their views and priorities changed in the same population as a result of the impacts of an intervention to alleviate the pressures of fuel poverty. The longitudinal approach allows insight into the change experienced following the intervention.^45^

In order for policy makers to assess the impacts of energy efficiency interventions on recipients, and to understand how to best target future policies that aim to alleviate fuel poverty, it becomes important to understand the views and experiences of householders and to understand the impacts of the intervention on individuals. Focus group participants in our study were generally positive about the energy efficiency programme and felt that such policy-led schemes are important for communities, such as theirs, but had some criticism about the overall aims and objectives and the delivery of the programme. This adds weight to the need to consider improved engagement and communication to involve residents more closely in the decision-making and delivery of affordable warmth programmes. Quantitative data allow for statistical understanding of the health and social impacts of an intervention but only for the limited questions asked by researchers, with the key aim of hypotheses testing. Therefore, it is also important to consider collecting qualitative data within a wider, mixed methods study to better know what are the breadth of views and the experiences and priorities of recipients, in order to understand how to improve future policy-led programmes. Relying on other data collection methods would not have allowed the exploration of the depth of issues and impacts on residents, which the longitudinal focus groups approach has enabled.

## Availability of data and material

The datasets analysed during the current study are available from the corresponding author on reasonable request.
